# Ghrelin Bridges DMV Neuropathology and GI Dysfunction in the Early Stages of Parkinson's Disease

**DOI:** 10.1002/advs.202203020

**Published:** 2022-09-01

**Authors:** Yizhen Liu, Weiwei Wang, Ning Song, Lingling Jiao, Fengju Jia, Xixun Du, Xi Chen, Chunling Yan, Jianwei Jiao, Qian Jiao, Hong Jiang

**Affiliations:** ^1^ Department of Physiology Shandong Provincial Key Laboratory of Pathogenesis and Prevention of Neurological Disorders and State Key Disciplines: Physiology, School of Basic Medicine Qingdao University Qingdao Shandong 710061 China; ^2^ State Key Laboratory of Stem Cell and Reproductive Biology Institute of Zoology Chinese Academy of Sciences Beijing 100101 China

**Keywords:** dorsal motor nucleus of vagus nerve, gastrointestinal dysfunction, ghrelin, Parkinson's disease

## Abstract

Ghrelin contributes to the communication between the brain and gastrointestinal (GI) tract. Both decreased ghrelin levels and functional GI disorders are early events in Parkinson's disease (PD) patients and animal models. However, the reason is not clear. Here it is found that choline acetyltransferase (ChAT)‐positive neurons in the dorsal motor nucleus of the vagus nerve (DMV), are lost in PD transgenic mice. In response to the selective damaging of DMV neurons with mu p75‐SAP, a rapid reduction both in plasma total and active ghrelin levels is observed. While by contrast, chemogenetic activation of DMV cholinergic neurons can increase the plasma ghrelin levels. Impairment of cholinergic neurons is accompanied by GI disorders, including decreased stool wet weight, stool dry weight, small intestine advancing rate, and gastric emptying rate, while exogenous ghrelin treatment can partially ameliorate GI dysfunction of A53T *α*‐synuclein transgenic mice. Using pseudorabies virus retrograde trace method, the existence of a direct pathway from the stomach fundus to the DMV is shown. Taken together, the findings suggest that the reduction in plasma ghrelin levels in the early stages of PD may be the result of the lesion of cholinergic neurons in the DMV, thus linking neurodegeneration and GI dysfunction in PD.

## Graphical abstract

Loss of cholinergic neurons in the dorsal motor nucleus of the vagus nerve is observed in Parkinson's disease transgenic mice models. Plasma ghrelin levels can be influenced via manipulating these neurons, and associated with gastrointestinal motility. Ghrelin, a well‐known brain‐gut peptide, established the communications between neurodegeneration and gastrointestinal dysfunction in Parkinson's disease.

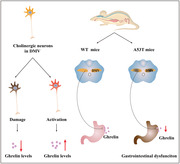

## 1 Introduction

Nonmotor symptoms may be present before the diagnosis of Parkinson's disease (PD).^[^ ^1^ ^]^ Gastrointestinal (GI) dysfunction, for example, is one of the commonest premotor symptoms reported around 60–80% of the PD patients.^[^ ^2^ ^]^ As a brain‐gut hormone, ghrelin has been proved to regulate GI function.^[^ ^3^ ^]^ In our previous study, we demonstrated that plasma total and active ghrelin levels were decreased in PD patients and mice.^[^ ^4^ ^]^ Combined with our previous evidences that GI dysfunction appeared before the loss of nigral dopaminergic neurons,^[^ ^5^ ^]^ ghrelin might be considered as a biomarker for GI dysfunction in early PD.

Extrinsic, preganglionic cholinergic fibers originating from the dorsal motor nucleus of the vagus nerve (DMV) are part of the enteric nervous system (ENS). The latter regulating GI motor, secretary, and circulatory activities. In sporadic PD, a six‐stage system (Braak) predicts a sequence of lesions with ascending progression from medullary and olfactory nuclei in the first two nonmotor stages, whereas motor symptoms appear at stages 3 and 4.^[^ ^6^ ^]^ Neuropathological studies of PD also support these two early stages by demonstrating the presence of Lewy body or Lewy neurites in the ENS and the DMV.^[^ ^7^ ^]^ Therefore, the neuropathological changes in the DMV might affect the GI function in PD. However, the role of ghrelin in the communication between the DMV neuropathology and GI dysfunction is largely unknown.

In the present study, plasma ghrelin levels were detected during the disease progression in PD transgenic mice expressing mutant A53T human *α*‐synuclein (*α*‐syn) (A53T mice). Interestingly, early loss of cholinergic neurons in the DMV was observed in A53T mice, which was accompanied by reduced plasma ghrelin levels and GI dysfunction. Using a pseudorabies virus (PRV)‐based retrograde tracing method, we also identified a direct nerve fiber connection between the mouse stomach and DMV. Our results might provide clues that GI dysfunction in PD might be due to the decreased plasma ghrelin levels. This connection could have implications for the neuropathologies in central nervous system in PD.

## 2 Results

### 2.1 Alterations of Plasma Ghrelin Levels and Total/Cholinergic Neurons in the DMV in Different Age Groups of A53T Mice

**Figure 1:**
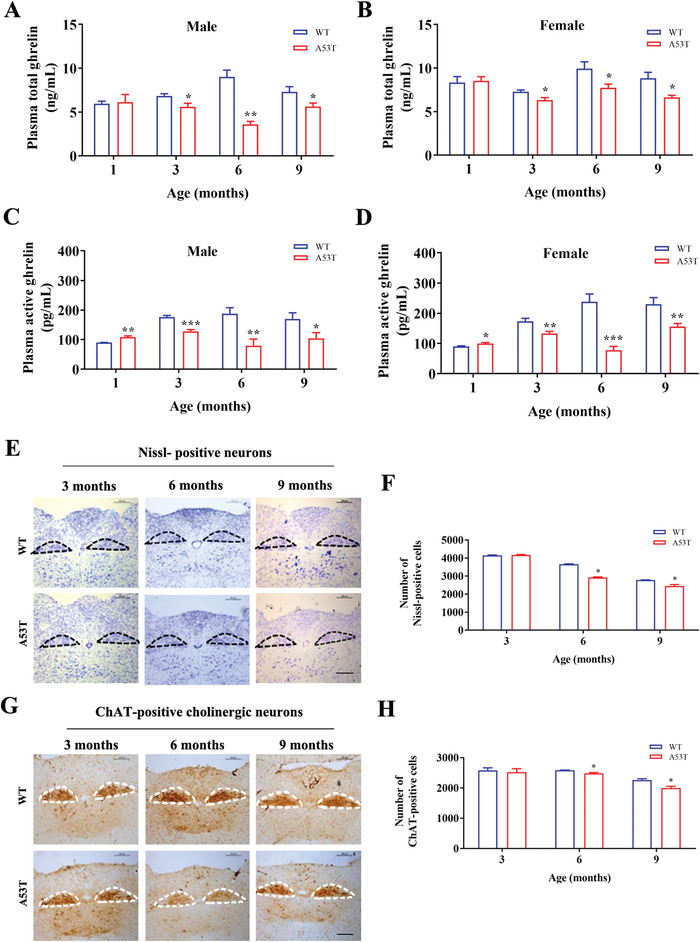
Changes of plasma ghrelin levels and cholinergic neurons in the DMV in heterozygous A53T and WT mice at various ages. A,B) Changes in the total plasma ghrelin levels changes of heterozygous A53T mice at different ages compared with that of their WT littermates. Statistical analysis was a Two‐way ANOVA. A) Df = 1, F = 11.3, *P* = 0.0012, for genotype; Df = 3, F = 0.09424, *P* = 0.963, for age. B) Df = 1, F = 6.864, *P* = 0.0108, for genotype; Df = 3, F = 3.384, *P* = 0.0228, for age, followed by *t*‐test between A53T and WT at each age. C,D) The Active plasma active ghrelin levels changes of heterozygous A53T mice at different ages compared with that of their WT littermates. Statistical analysis was a Two‐way ANOVA. C) Df = 1, F = 10.59, *P* = 0.0019, for genotype; Df = 3, F = 1.622, *P* = 0.1943 for age. D) Df = 1, F = 28.94, *P* < 0.0001, for genotype. Df = 3, F = 9.825, *P* < 0.0001, for age, followed by *t*‐test between A53T and WT at each age. E) Representative images of Nissl‐positive cells that stained by Nissl of male heterozygous A53T mice at various ages. Encircled areas: DMV region. Scale bar: 100 µm. F) Statistical analysis of Nissl‐positive cells of male heterozygous A53T mice. Statistical analysis was a Two‐way ANOVA (Df = 1, F = 106, *P* < 0.0001, for genotype; Df = 2, F = 690, *P* < 0.0001, for age), followed by *t*‐test between A53T and WT at each age. G) Representative images of ChAT‐positive cells that stained by ChAT antibody of male heterozygous A53T mice at various ages. Encircled areas: DMV region. Scale bar: 100 µm. H) Statistical analysis of ChAT‐positive cells that stained by ChAT antibody of male heterozygous A53T. Statistical analysis was a Two‐way ANOVA (Df = 1, F = 6.584, *P* = 0.0247, for genotype; Df = 2, F = 24.50, *P* < 0.0001, for age), followed by *t*‐test between A53T and WT at each age. All data were presented as mean ± SEM (*t*‐test, *P < 0.05, **P < 0.01, ***P < 0.001, A53T vs WT littermates at the same age), *n* ≥ 6 for each group.

To evaluate whether changes in the plasma ghrelin levels were an early event during the progression of PD, we measured plasma total and active ghrelin levels in both WT and heterozygous A53T mice. Two‐way ANOVA statistical analysis revealed the difference between genotypes or among ages for both plasma total and active ghrelin levels in female mice, while only the difference between genotypes was observed in male mice (*P* < 0.05). Plasma total ghrelin levels were both reduced in 3‐, 6‐, and 9‐month‐old heterozygous male and female A53T mice, compared with that of age‐matched controls (**Figure** **1**A,B). In both genders, plasma active ghrelin levels were decreased in heterozygous A53T mice at 3, 6, and 9 months of age; however, the A53T mice at 1 month of age exhibited a 10% increase (Figure 1C,D). Next, we sought to determine the central regulatory mechanism underlying the decreased plasma ghrelin levels. We calculated the total number of Nissl‐positive neurons and choline acetyltransferase (ChAT)‐positive cells in the DMV of 3‐, 6‐, and 9‐month‐old heterozygous A53T and WT littermates. Two‐way ANOVA statistical analysis showed that significant differences between genotypes or among ages on the effects of Nissl‐positive neurons loss and ChAT‐positive cells loss in male mice, respectively (*P* < 0.05). Male heterozygous A53T mice exhibited a no significant difference relative to control at the age of 3 months (Figure 1E,F). In 6 and 9‐month‐old males, the total number of Nissl‐positive cells in the DMV was lower than that of the corresponding WT controls (Figure 1E,F). In parallel with the data we obtained for Nissl‐positive neurons, the total number of ChAT‐positive cells in the DMV of male mice was lower at 6 and 9 months of age, respectively (Figure 1G,H). Female A53T mice did not exhibit a significant difference in the Nissl‐positive cell count until 9 months, at which point a 12.46% reduction was observed (Figure S1A,B, Supporting Information). Females exhibited a 12.30% reduction in the total number of ChAT‐positive cells at 9 months of age (Figure S1C,D, Supporting Information). Our findings suggested that cholinergic neurons in the DMV of heterozygous A53T mice were impaired at the early stage of PD.

### 2.2 Plasma Ghrelin Levels were Regulated by DMV Cholinergic Neurons

**Figure 2:**
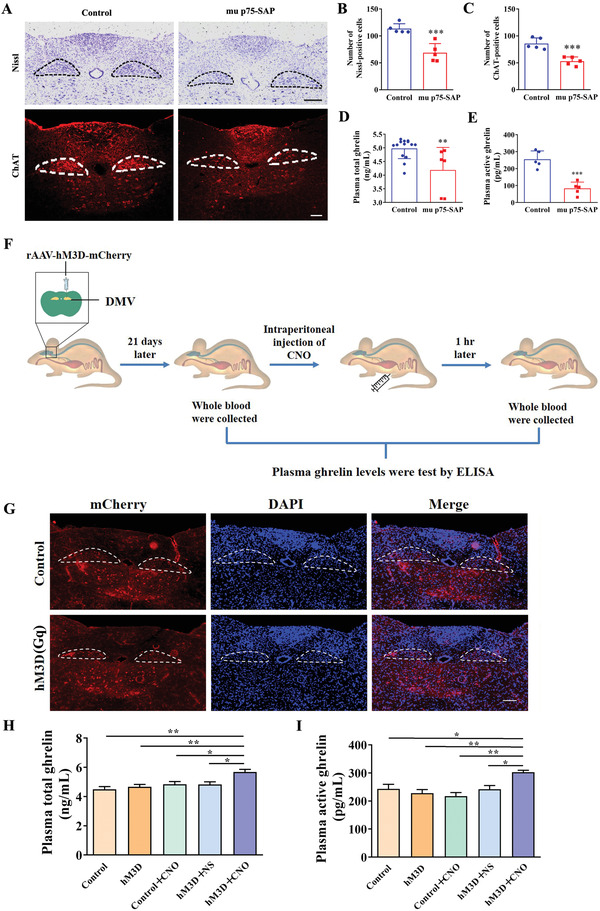
Changed plasma ghrelin levels depended on the activation of cholinergic neurons in the DMV. A) Representative images of Nissl‐positive cells and ChAT‐positive cells that stained by ChAT antibody of mice both in control and mu p75‐SAP‐treated group. Encircled areas: DMV region. Scale bar: 100 µm. B) Statistical analysis of Nissl‐positive cells. C) Statistical analysis of ChAT‐positive cells. D) Total plasma ghrelin levels were decreased in mu p75‐SAP‐treated mice group. E) Plasma active ghrelin levels were decreased in mu p75‐SAP‐amdinistered animals group. F) Schematic diagram of chemical genetic experiments. To assess if cholinergic DMV neurons are responsible for the secretion of ghrelin, cholinergic neurons in the DMV were infected by mCherry‐tagged hM3D (Gq) virus, which could be activated by CNO. The plasma ghrelin levels were analyzed before and after CNO injection. G) Expression of mCherry was observed in DMV region of ChAT‐cre mice with mCherry‐tagged virus injection. Encircled areas: DMV region. Scale bar: 100 µm. H) Effects of activation of cholinergic neurons in DMV on total plasma ghrelin levels. I) Effects of activation of cholinergic neurons in the DMV on active plasma ghrelin levels. B–E) Data were mean ± SEM (*t*‐test, **P < 0.01, ***P < 0.001, compared with the control), *n* ≥ 5 for each group. H,I) Data were mean ± SEM (One‐way ANOVA, followed by the Tukey test, *P < 0.05, **P < 0.01), *n* ≥ 5 for each group.

To investigate the relationship of DMV and ghrelin secretion, mu p75‐SAP was used to elicit excitotoxic lesions in C57BL/6 mice, which resulted in spatially controlled neuronal damage.^[^ ^8^ ^]^ Nissl‐positive cells and ChAT‐positive cholinergic neurons (**Figure** **2**A) in mu p75‐SAP‐treated group were lower than that of the corresponding controls, respectively (Figure 2B,C). Compared with the control group, the total and active plasma ghrelin levels of mu‐p75 SAP‐treated group were decreased by 16% and 67%, respectively (Figure 2D,E).

Chemical genetics provide an effective way to modulate neuronal activity to study the function of selected neurons. To further confirm the relationship between DMV and ghrelin secretion, we used chemical genetics methods to activate the cholinergic neurons of ChAT‐Cre mice (Figure 2F). The rAAV‐Ef1*α*‐DIO‐hM3D (Gq)‐mCherry (abbreviated as: hM3D (Gq)) virus was administered into the medulla oblongata DMV area of ChAT‐Cre mice, and the whole blood was collected before and after clozapine‐N‐oxide (CN) injection (Figure 2F). CNO could specifically activate the neurons with hM3D (Gq) expression. Twenty‐one days after infection, ChAT‐positive cells transgenically expressing mCherry‐tagged hM3D (Gq) were detected in DMV of mice (Figure 2G). After 1 h of intraperitoneal injection of CNO, levels of plasma total ghrelin and active ghrelin in hM3D (Gq) +CNO group mice were increased, which were higher than that of Control, hM3D (Gq), Control +CNO, and hM3D (Gq) +NS groups, respectively. Additionally, there was no difference among Control, hM3D (Gq), Control +CNO, and hM3D (Gq) +NS groups (Figure 2H,I). These results suggested that cholinergic neurons in the DMV regulated plasma ghrelin levels.

### 2.3 GI Function Disorders with DMV Lesions

**Figure 3:**
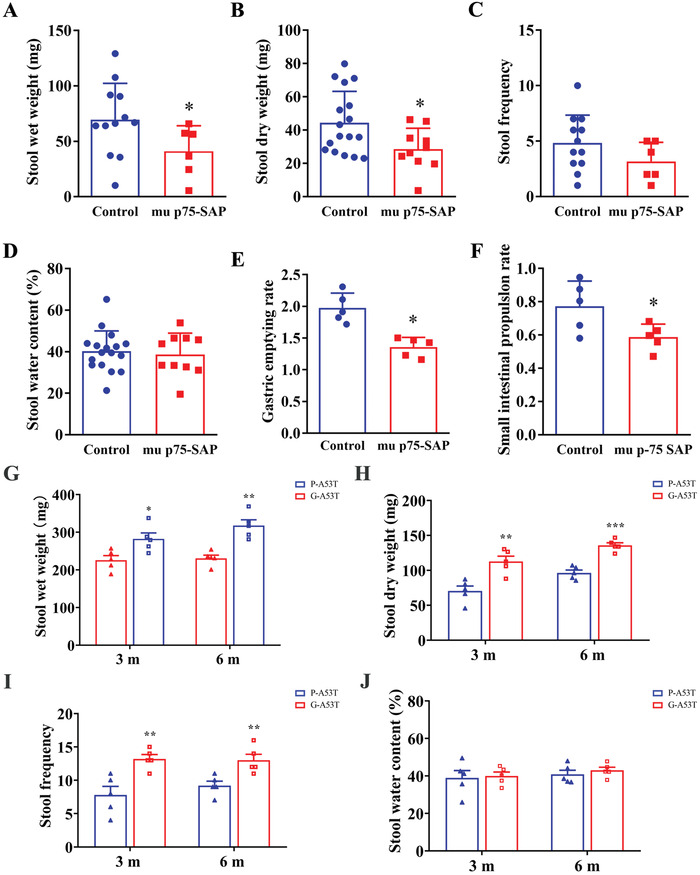
Effects of the cholinergic neurons lesion in the DMV on gastrointestinal motility in mice. Various gastrointestinal markers were assessed in WT mice with mu p75‐SAP‐ treatment, or in A53T mice with ghrelin (G‐A53T) or scrambled ghrelin peptide (P‐A53T). A,G) Stool wet weight. B,H) Stool dry weight. C,I) stool frequency. D,J) Stool water contents. E) Small intestinal motility. F) Gastric emptying rate. A–F) All data were mean ± SEM (*t*‐test, *P < 0.05, compared with the control), *n* ≥ 5 for each group. G–J) All data were mean ± SEM (*t*‐test, *P < 0.05, **P < 0.01, ***P < 0.001, G‐A53T vs P‐A53T at the same time point), *n* = 5 for each group.

To determine the regulatory function of cholinergic DMV neurons in the GI tract, the medulla oblongata DMV area of mice was lesioned with mu p75‐SAP. After 14 days of mu p75‐SAP treatment, the wet weight and dry weight of feces were decreased by 35% and 36%, respectively, compared with that of the corresponding controls (**Figure** **3**A,B). The stool frequency and water contents of feces were unchanged compared with the control group (Figure 3C,D). The small bowel advancement rate was decreased by 24% (Figure 3E) and the gastric emptying rate was decreased by 31% (Figure 3F) in comparison with those of the corresponding controls. These findings showed that impaired cholinergic neuron function in the DMV resulted in GI disorder.

In order to confirm the regulation of ghrelin on GI functions, we administered low and continuous doses of ghrelin to sustain its concentration via spontaneous *Alzet* miniosmotic pumps before the onset of plasma ghrelin decrease and then detected whether recovered ghrelin could ameliorate GI dysfunction of A53T mice. A53T mice were treated with ghrelin (G‐A53T mice) and scrambled ghrelin peptide (P‐A53T mice) for 8 weeks, respectively. Results showed that the stool wet weight of G‐A53T mice was higher than that of corresponding control P‐A53T mice both at 3 and 6 m (Figure 3G). Moreover, the stool dry weight of G‐A53T mice was also higher than that of P‐A53T mice at 3 and 6 m, respectively (Figure 3H). Stool frequency were upregulated on G‐A53T mice compared with that of P‐A53Tmice at 3 and 6 m, respectively (Figure 3I). We also found there were no differences in stool water contents between G‐A53T mice and P‐A53T mice at each test time points (Figure 3J). These findings showed that ghrelin treatment could partially ameliorate some GI dysfunction of A53T mice.

### 2.4 Identification of a Direct Neural Connection from DMV to Stomach

**Figure 4:**
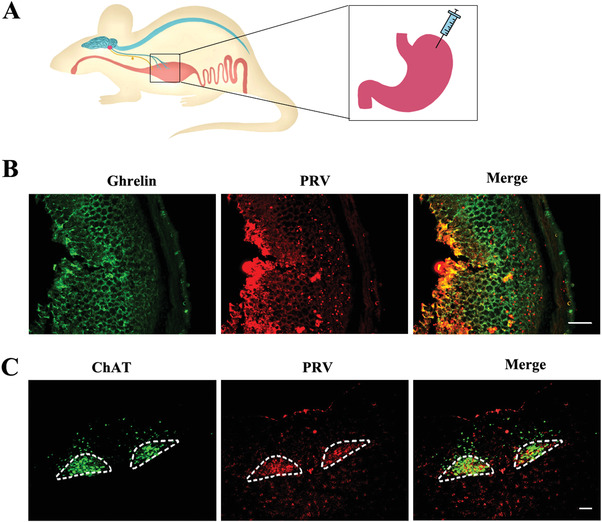
Retrograde tracing of nerve fibers from the gastric fundus to the DMV region. A) Model of injection of PRV‐CAG‐EGFP virus in the gastric fundus. B) Co‐localization of PRV‐CAG‐EGFP expression and was observed on the ghrelin‐positive cells (stained by ghrelin antibody). Scale bar: 100 µm, *n* = 5. C) GFP was observed on the ChAT‐positive neurons that stained by ChAT antibody in the DMV area. Encircled areas: DMV region. Scale bar: 100 µm, *n* = 5.

In order to prove that neuronal projections from the DMV regulate the release of ghrelin via the gastrointestinal tract through neuronal projections, we observed the retrograde transmission of PRV from the gastric fundus to the DMV using retrograde tracer technique. After 3 days of injection of pseudorabies virus containing *GFP* in the gastric fundus of C57BL/6 male mice (**Figure** **4**A), we observed the coexpression of ghrelin‐positive cells and GFP in the stomach fundus (Figure 4B) and coexpression of ChAT‐positive cells and GFP in the medulla oblongata DMV area (Figure 4C). Thus, there existed a direct nerve fiber pathway from stomach to DMV.

## 3 Discussion

Ghrelin, a brain‐gut peptide, is primarily released by X/A‐like cells of the fundus of stomach. One important roles of ghrelin in the brain‐gut axis is to regulate food intake, body weight and blood glucose.^[^ ^9^ ^]^ Recently, it was reported that GI dysmotility is associated with impaired ghrelin signaling in gastroesophageal reflux disease, and exogenous ghrelin treatment could increase antral motility.^[^ ^10^ ^]^ In the previous study, we reported that plasma ghrelin levels, both total and active (acyl‐) form, were decreased in PD patients and homozygous A53T mice.^[^ ^4^ ^]^ The early GI abnormalities were recapitulated in both A53T and A30P transgenic mouse models by 3 months of age in the absence of dopaminergic neurodegeneration.^[^ ^5^, ^11^ ^]^ Robust abnormalities in ENS function and alpha‐synuclein‐immunoreactive aggregates in ENS were shown in these models, however, more recently, extensive *α*‐synuclein deposits were observed in the cholinergic efferent vagus nerve fibers, rather than enteric neurons.^[^ ^12^ ^]^ GI motility remained unaltered, suggesting that ghrelin regulation and the corresponding local neuronal network were normal at this early stage of these models. This result was further supported by a postmortem study in PD patients, which demonstrated that GI dysfunction in PD was unlikely to be related to intrinsic myenteric neuronal pathology.^[^ ^13^ ^]^ Consistent with this result, we did observe a decrease in circulating ghrelin levels (Figure 1A–D). This evidence led us to investigate the more dominant central mechanisms controlling ghrelin secretion.

Ghrelin is not only an important brain‐gut peptide in the regulation of gastrointestinal movements, but also has nonmetabolic functions, such as neuroprotection and the regulation of inflammatory response. However, all these functions depended on the integrity of vagus nerve.^[^ ^14^ ^]^ For example, evidences showed that ghrelin resistance in high fat diet‐fed mice may be caused by the reductions of growth hormone secretagogue receptor type 1*α* mRNA levels in the nodose ganglion of the vagus nerve, caused by local and hypothalamic which related to inflammatory responses occurred in the nodose ganglion and hypothalamus.^[^ ^15^ ^]^ It is also established that obese animals show reduced plasma ghrelin levels.^[^ ^16^ ^]^ Finally, the intracerebroventricular injection of ghrelin treatment protected from sepsis‐evoked gut dysfunction in vivo, was only in the present of vagus nerve activation.^[^ ^17^ ^]^ Parasympathetic motor neurons in the DMV innervate the stomach via vagal nerve branching, which indicates that this connection might regulate GI function.^[^ ^18^ ^]^ Using PRV retrograde tracing in this study, we confirmed that such a direct connection exists (Figure 4). Therefore, we are particularly interested in whether DMV dysfunction contributes to impaired ghrelin secretion. Furthermore, as expected, both Nissl‐positive and ChAT‐positive cell numbers were reduced in 6‐ and 9‐month‐old male heterozygous A53T mice (Figure 1E–H). These results suggested that the loss of ChAT expressing positive motor neurons in the DMV might contribute to the decreased secretion of ghrelin via the vagus nerve during PD progression. The hypothesis was further supported by our findings that a unilateral DMV lesion induced by mu p75‐SAP caused a rapid reduction of total and active plasma ghrelin levels, indicating disrupted control of ghrelin secretion (Figure 2A–E). Accordingly, movements of GI tract including stool wet weight, stool dry weight, small intestine advancing rate, and gastric emptying rate, were changed after mu p75‐SAP treatment (Figure 3A–F). This supported that DMV influence gastrointestinal function through ghrelin. Moreover, activation of the cholinergic neurons in the DMV caused an increase of ghrelin secretion (Figure 2G–H), while exogenous ghrelin treatment with physiological concentrations improved the GI function (Figure 3G–J). In our previously study, A53T mice suffered from the GI function of at age of 3 month.^[^ ^5^ ^]^ However, the GI parameters such as lower stool wet weight, dry weight and frequency observed in the chemically lesioned control groups corresponding to mu p75‐SAP treatment were lower than that of A53T mice, which might be due to the surgical operations in the DMV. Notably, plasma ghrelin levels were decreased in A53T mice at 3 months of age, although no difference of ChAT‐positive motor neurons and total neurons were observed in A53T versus their WT littermates (Figure 1A). This result might be due to the functional damage to cholinergic neurons that regulate ghrelin secretion in the DMV of A53T mice. Interestingly, the number of DMV neurons appeared to be unaffected in female A53T mice at 6 months of age and decreased in age‐matched males. The delayed neurodegeneration may be associated with the neuroprotective effects of estrogen.^[^ ^19^ ^]^ Collectively, the results of this study warrant to evaluate plasma ghrelin levels as a biomarker in the preclinical stages of PD.

## 4 Conclusion

Taken together, our data suggest a modest reduction in plasma ghrelin levels in the A53T PD mouse model, presumably resulting from impaired DMV function. We propose that the reduction in ghrelin levels is related to the GI symptoms in PD from the lesioned DMV, thus establishing a communications between neurodegeneration and GI dysfunction. These results also provide the evidence for ghrelin as an early diagnosis biomarker for PD.

## 5 Experimental Section

### Animals

All experimental procedures were performed in accordance with the National Institutes of Health Guide for the Care and Use of Laboratory Animals and approved by the Ethical Committee of Medical College of Qingdao University (NO: QDU‐AEC‐2022311).

Transgenic mice expressing mutant A53T human *α*‐syn driven by the prion promoter were obtained from the Jackson Laboratory.^[^ ^20^ ^]^ Experiments were performed on heterozygous mice. Mice were evaluated at multiple ages: 1, 3, 6, and 9 months. C57BL/6 male mice used in this study were purchased from Wei Tong Li Hua (Beijing, CN). ChAT‐Cre mice were kindly provided by Professor Jiawei Zhou in Shanghai Chinese Academy of Sciences.

Whole blood was collected from the inner canthus in the morning after being fasted overnight under deep anesthesia (sodium pentobarbitone, 45 mg kg^−1^).

### Measurement of Plasma Ghrelin Levels via Enzyme‐Linked Immunosorbent Assay (ELISA)

Whole blood was collected into EDTA‐plasma tubes. 4‐(2‐Aminoethyl) benzenesulfonyl fluoride hydrochloride (AEBSF) was immediately added (1 mg mL^−1^ final concentration), followed by centrifugation (20 min at 3000 × *g* at 4 °C). Plasma samples were acidified with HCl (0.05 mol L^−1^ final concentration). Active and total ghrelin levels in plasma were measured using commercially available immunoassay kits: mouse ghrelin (active) or mouse ghrelin (total) ELISA kits (EZGRA‐88K/EZRGRA‐90K or EZGRT‐89K/EZRGRT‐91K, respectively; Millipore) according to the manufacturer's instructions.

### Nissl Staining, Immunohistochemistry, and Immunofluorescence

Mouse brains and stomach were removed and postfixed in 4% paraformaldehyde at 4 °C. The brains were cryoprotected in 20% and 30% sucrose at 4 °C, sequentially. Sections from DMV were rinsed in distilled water for 2 min and stained with Nissl staining solution (Beyotime, CN) for 10 min at room temperature. Antibodies specific to ChAT (1:100, Millipore, USA), ghrelin (1:500, Phoenix, USA), horseradish peroxidase‐conjugated secondary antibody (1:1000, Santa Cruz, USA), Alexa 488‐conjugated secondary antibody (1:500, Invitrogen) and Alexa 555‐conjugated secondary antibody (1:500, Invitrogen) were used. Images were captured with a light microscope (Leica, German) and a fluorescence microscope (ZEISS, German). Nissl‐positive neurons and ChAT‐positive cholinergic neurons in the DMV were obtained using unbiased stereology according to the optical fractionators principle described before.^[^ ^21^ ^]^

The numbers of stained neurons was determined every sixth section covering the entire DMV for each mouse. For each section, the boundaries of the regions were first delineated at low magnification (×10) and counting was performed at high magnification (×40). The analysis was performed using MBF Stereo Investigator software (MBF Bioscience). Stereological details were as follows: DMV counting grid, 160 × 160 µm^2^; counting frames, 70 × 70 µm^2^.

### Ghrelin Treatments

The experimental protocols of ghrelin treatment via *Alzet* miniosmotic pumps (Alzet, USA) were performed as the previous study.^[^ ^4b^ ^]^ P‐A53T group: A53T mice with administered nonsense peptide (a scrambled ghrelin peptide; amino acid sequences: GLSFEHQSPQQRAKEKKSP KLPAQPRK); G‐A53T group: A53T mice with administered ghrelin (Sigma, USA) (GSSFLSPEHQKAQQRKESKKPPAKLQPR), which contained acylated ghrelin as physiological level. Ghrelin and nonsense peptide were administered subcutaneously in saline via *Alzet* miniosmotic pumps (Alzet, USA). There were two time points: 1) mice that were administered peptide starting at the age of 1 month, lasting for 8 weeks, and that were harvested at the age of 3 months (3 m); 2) mice that were administered peptide starting at the age of 1 month, lasting for 8 weeks, and that were harvested at the age of 6 months (6 m).

### Chemical Lesion of the DMV

Chemical lesion of the DMV was performed by mu p75‐SAP injection into the DMV to induce neurotoxicity.^[^ ^8^ ^]^ Briefly, C57BL/6 mice at 8–10 weeks of age were anesthetized, and bilateral lesions were conducted via the injection mu p75‐SAP (1.4 mg mL^−1^) in 0.2 µL using a Hamilton syringe (maximum effective range: 0.5 µL, needle size: 33G); saline alone was used as a control. An incision was made to expose the scalp, and one burr hole (2.5 mm diameter) was drilled into the skull above the DMV according to the coordinates of Paxinos and Watsons: AP, −7.32 mm; ML, 0.25 mm; DV, −4.35 mm. Blood samples were collected 2 h after mu p75‐SAP injection as described above.

### DMV Virus Administration

The adeno‐associated viral vector rAAV‐Ef1*α*‐DIO‐hM3D (Gq)‐mCherry and control virus (rAAV‐Ef1*α*‐DIO‐mCherry) were purchased from BrainVTA Co., Ltd (Wuhan, CN). ChAT‐Cre mice at 8–10 weeks of age were anesthetized, and 0.2 µL virus (2.0E+12 PFU mL^−1^) was bilateral injected into DMV by hanmilton syringe (33G). Twenty‐one days later, 300–500 µL whole blood were collected from the inner canthus, then animals were administrated by intraperitoneal injection of CNO (1 mg/mL) immediately. CNO can specifically activate the neurons which infected by rAAV‐Ef1*α*‐DIO‐hM3D (Gq) –mCherry. The whole blood was collected again after CNO injection for 1 hr. Following gradient elution by sucrose solutions, brain tissues were cut in 20 µm sections for immunohistochemical staining.

### Stomach Virus Administration

C57BL/6 mice at 8–10 weeks of age were anesthetized and fixed on operation platform. After sterilization, an incision with length of 1.5 cm was made along the middle of abdomen to expose stomach. PRV‐CAG‐EGFP (6.0E+09 PFU/mL) was injected to the fundus submucosa layer of stomach via Hamilton syringe in 0.6 µL (maximum effective range: 1 µL). Animals were continued feeding for 4 days after virus infection, then stomach and brain tissues were removed and fixed by 4% PFA. Following gradient elution by sucrose solutions, brain tissues were cut in 20 µm sections for immunohistochemical staining.

### Gastrointestinal Function Testing—Colonic Motility

1) One‐hour stool frequency assays: One‐hour stool frequency assay was performed between 10:00 and 12:00 daily. Animals were removed from the home cage and placed individually in a clean, clear plastic cage without food or water for 1 hour. Stools were collected immediately and placed in sealed tubes to calculate the sum for each individual (stool frequency).^[^ ^5^, ^12^ ^]^ 2) Stool weight: The total stools were weighed to provide a wet weight, then dried overnight at 65 °C and weighed again to provide a dry weight.^[^ ^5^, ^12^ ^]^

### Gastrointestinal Function Testing—Gastric Emptying Rate

Gastric emptying rate was measured by the food expulsion test. A mixture food contained H_2_O 125 mL, sodium carboxymethylcellulose 5 g, sucrose 4 g, starch 4 g, milk powder 8 g, activated carbon powder 1.5 g were made and stored at 4°C. Briefly, the mixture food in tube was inserted into the stomach with dose 0.025 mL/g by intragastric gavage after fasting for 18 hrs. Animals were killed 20 minutes after gavage. The pylorus and cardia were ligated by surgical suture, and then the stomach was removed and weighed (as m1). Then cut stomach along the greater curvature, washed with saline to clear out the gastric contents, and weighed stomach (as m2). The formula of gastric emptying rate was = 1‐(m1‐m2)/total weight of food in the stomach.

### Gastrointestinal Function Testing—Intestinal Propulsive Rate

The intragastric gavage administration was followed as above. Small intestine from pylorus to cecum were dissociated and removed. The total length of small intestine (as L1) and the distance that from pylorus to small intestine where reserved food were measured (as L2). The formula of intestinal propulsive rate was = L2/L1.

### Statistical Analysis

Results were presented as means ± SEM. The sample size (n) in all groups that used for statistical analysis was more than 5. All data were analyzed by the SPSS 19.0 software and GraphPad Prism 8. Differences between means in two groups were compared using the unpaired‐samples *t* test. One‐way analysis of variance (ANOVA) followed by the Tukey test was used to compare differences between means in more than two groups. The results from plasma ghrelin levels, Nissl‐positive cells and ChAT‐positive cells assessments were analyzed using Two‐way ANOVA followed by t‐test between A53T and WT at each age. A probability of *P* < 0.05 was used to indicate statistical significance.

## Conflict of Interest

The authors declare no conflict of interest.

## Author Contributions

Y.L., W.W., and N.S. contributed equally to this work. Y.L., W.W., and N.S. performed the major experiments. L.J. performed the ghrelin treatments via miniosmotic pumps on A53T mice. N.S. analyzed data in A53T mice. F.J. and C.Y. contributed to experiments in mice. N.S., X.D., X.C., and J.J. helped to write the manuscript. Q.J. and H.J. conceived the project, designed experiments, supervised the whole project and wrote the manuscript.

## Supporting information

Supporting Information

## Data Availability

The data that support the findings of this study are available from the corresponding author upon reasonable request.
