# Supplementary material for: Ghrelin Bridges DMV Neuropathology and GI Dysfunction in the Early Stages of Parkinson's Disease
Source: Adv Sci (Weinh). 2022 Sep 1;9(30):2203020. doi: 10.1002/advs.202203020 (PMC9596842; doi:10.1002/advs.202203020)
Supplement: Supplementary file 1 — Supporting Information [file ADVS-9-2203020-s001.pdf]

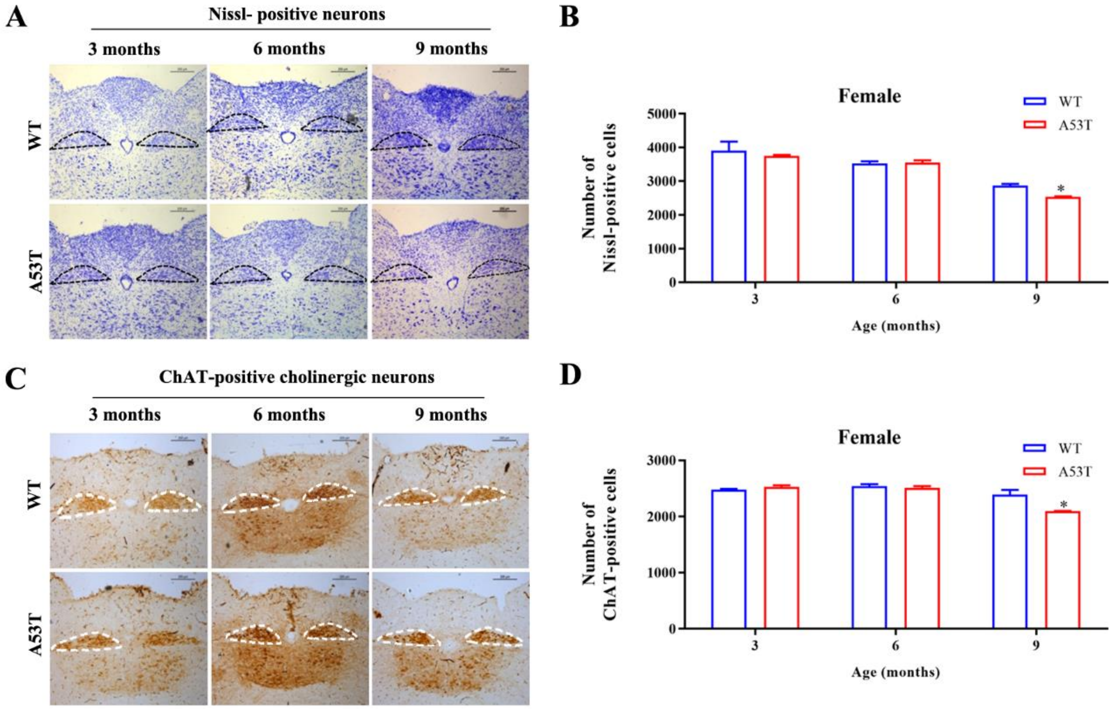

S1. Neurons loss in the DMV in heterozygous female A53T and WT mice at various ages. A: Representive images of Nissl-positive cells that stained by Nissl of female heterozygous A53T mice at various ages. Encircled areas: DMV region. Scale bar: 100 μm. **B**: Statistical analysis of Nissl-positive cells of female heterozygous A53T mice. Statistical analysis was a Two-way ANOVA (Df =1, F=2.642, P=0.13, for genotype; Df =2, F=48.24, P<0.0001, for age), followed by t-test between A53T and WT at each age. **C**: Representive images of ChAT-positive cells that stained by ChAT antibody of female heterozygous A53T mice at various ages. Encircled areas: DMV region. Scale bar: 100 μm. **D**: Statistical analysis of ChAT-positive cells that stained by ChAT antibody of female heterozygous A53T. Statistical analysis was a Two-way ANOVA (Df =1, F=7.149, P=0.0223, for genotype; Df =2, F=28.47, P<0.0001, for age), followed by t-test between A53T and WT at each age. All data were presented as mean ± SEM. (t-test, $^{*}P < 0.05$, A53T vs. WT littermates at the same age), n ≥ 6 for each group.
